# Outcomes Assessment of Sustainable and Innovatively Simple Lifestyle Modification at the Workplace-Drinking Electrolyzed-Reduced Water (OASIS-ERW): A Randomized, Double-Blind, Placebo-Controlled Trial

**DOI:** 10.3390/antiox9070564

**Published:** 2020-06-27

**Authors:** Young Ah Choi, Dong Hyeon Lee, Doo-Yeoun Cho, Yong-Jae Lee

**Affiliations:** 1 Department of Family Medicine, Bundang Jesaeng General Hospital, Seongnam-si, Gyeonggi-do 13590, Korea; 2 Department of Physiology, School of Medicine, CHA University, Seongnam-si, Gyeonggi-do 13488, Korea; leedh@cha.ac.kr; 3 Department of Clinical Pharmacology, School of Medicine, CHA University, Seongnam-si, Gyeonggi-do 13496, Korea; dooycho@cha.ac.kr; 4 Department of Family Medicine, Yonsei University College of Medicine, Seoul 06273, Korea; ukyjhome@yuhs.ac

**Keywords:** oxidative stress, electrolyzed-reduced water, biomarkers of oxidative stress, reactive oxygen metabolites, biological antioxidant potential, fat mass, lifestyle modification

## Abstract

Oxidative stress has been associated with many diseases as well as aging. Electrolyzed-reduced water (ERW) has been suggested to reduce oxidative stress and improve antioxidant potential. This study investigated the effects of drinking ERW on biomarkers of oxidative stress and health-related indices in healthy adults. We conducted a randomized, double-blind, placebo-controlled clinical trial on 65 participants, who were allocated into two groups. Of these, 61 received intervention (32 with ERW and 29 MW [mineral water]). All participants were instructed to drink 1.5 L/day of ERW or MW for eight weeks. Biomarkers of oxidative stress and health-related indices were assessed at baseline as well as after 4 weeks and 8 weeks of intervention. Of the primary outcome variables assessed, diacron-reactive oxygen metabolites (d-ROMs) and biological antioxidant potential showed a significant interaction between the groups and time, with d-ROMs levels significantly decreased at 8 weeks in ERW compared to those in MW. Among the secondary outcome variables, total, visceral, and subcutaneous fat mass significantly changed over time, with a significant association observed between the group and time. Thus, daily ERW consumption may be a potential consideration for a sustainable and innovatively simple lifestyle modification at the workplace to reduce oxidative stress, increase antioxidant potential, and decrease fat mass.

## 1. Introduction

Free radicals were first described in 1950 [1] and refer to highly unstable and reactive atoms or molecules, owing to unpaired electrons found in their outer orbit [2]. Reactive oxygen species (ROS) include reactive nonradical derivatives of oxygen in addition to oxygen-centered radicals [2]. Reactive species are produced not only by natural biological processes but are also generated in response to external stimuli, such as air pollution, ultraviolet (UV) radiation, infections, inflammation, heavy metals, cigarette smoking, drugs, strenuous exercise, and emotional stress [3,4]. Oxidative stress [5] generated from a disturbance in the balance between the formation and removal of reactive species has been shown to result in oxidative damage to various macromolecules, such as lipids, DNA, and proteins [2,5,6,7,8], and have been implicated as an etiological agent in many pathologic processes including aging [2,6,9,10], radiation injury, cardiovascular disease, diabetes, atherosclerosis, neurological diseases, and cancers [3]. Endogenous antioxidant defense mechanisms act together with exogenous antioxidants to prevent oxidative damage. Our body has an endogenous defense mechanism, including enzymatic and non-enzymatic systems, to counteract oxidative stress. Hence, diverse strategies exist for estimating oxidative stress status, including detection of free radicals, ROS, damaged macromolecules from oxidative stress, antioxidant capacity, and antioxidant enzymes for antioxidant potential, as well as health-related indices for altered medical conditions resulting from oxidative stress [7,11].

Electrolyzed-reduced water (ERW) is produced near the cathode during water electrolysis [12]. Accordingly, ERW has been shown to be rich in hydrogen atoms and hydrogen molecules, low in dissolved oxygen, with an alkaline pH, and has negative oxidation-reduction potential (ORP) [13,14]. An increasing number of studies have been conducted on alkaline and ERW since the effectiveness of ERW at scavenging ROS in vitro as well as its ability to protect against oxidative stress-induced DNA damage [15].

ERW has been shown to suppress oxidative stress by improving the function of antioxidant enzymes in U937 cell line [16], protect neural cells from oxidative damage [17], inhibit tumor angiogenesis [18], enhance apoptosis of leukemia cells (HL-60) [12,19], shorten cancer cell telomeres [14,20], and suppress invasion of human fibrosarcoma HT-1080 cells [21]. There was also a noteworthy study after the Fukushima disaster in 2011, suggesting that radionuclides, such as cesium and iodine could be removed by an ERW-producing apparatus [22]. In animal studies, ERW was effective in lowering the levels of blood glucose in both type 1 and type 2 diabetic mice models [23], lowering plasma triglycerides levels and suppressing lipid peroxidation levels [24], reducing lipopolysaccharide (LPS)-induced neuroinflammation [25], demonstrating neuroprotection in cisplatin-induced kidney damage and improving oxidative stress biomarker levels [26], as well as prolonging the lifespan of nematodes [27] and mice [28]. Similarly, ERW was reported to reduce oxidative stress [29] and erythrocyte impairment [30] while improving T-cell damage [31] in patients with end-stage renal disease undergoing hemodialysis.

Meanwhile, natural reduced water found in various natural environments, such as deep underground reservoirs and springs, and known to scavenge ROS [12], has been reported to inhibit alloxan-induced β-cell apoptosis [32], lower glucose levels in alloxan-induced mice [32], suppress anxiety in rats [33], and increase the activity of natural killer cells in healthy volunteers [34]. Moreover, drinking alkaline MW was shown to lower the bone resorption marker in adults with sufficient calcium [35] and to significantly decrease the concentration of lactic acid at rest in patients in a clinical trial [36]. In an open-label pilot study, consumption of hydrogen-rich water supplemented with a mineral stick improved indices related to oxidative stress and metabolic syndrome [37].

Health promotion programs adopting lifestyle modifications at the workplace are being implemented in several countries [38]. Several randomized clinical trials have been conducted to assess the effects of these healthy behaviors at the workplace, some of which reported meeting their primary endpoint, while others did not [39,40,41,42]. Nevertheless, these trials have raised questions regarding issues related to sustainability, affordability, and cost-effectiveness in terms of public and planetary health [38,43]. Daily consumption of water with improved function in the workplace may elicit a more positive effect on health promotion. However, an insufficient number of randomized, double-blinded, placebo-controlled clinical trials have investigated the effects of drinking ERW on healthy workers’ oxidative stress levels and related health. Therefore, the objective of this study was to assess the effects of sustainable and innovatively simple lifestyle modification at the workplace, namely drinking ERW for eight weeks, on biomarkers of oxidative stress and health-related indices in healthy adults.

## 2. Materials and Methods

### 2.1. Overview of the Study Design

We conducted a single-center, double-blind, placebo-controlled, parallel-group study in South Korea, in accordance with the “Ethical principles for medical research involving human subjects” defined in the declaration of Helsinki in 1975 (last updated in 2018), and following the Consolidated Standards of Reporting Trials (CONSORT) guidelines. The institutional review board of Bundang Jesaeng Hospital in Korea approved the study (IRB DMC 2018-11-009), and all subjects provided informed consent prior to participation. The study was registered at the Clinical Research Information Service-Republic of Korea (CRIS, https://cris.nih.go.kr/cris/) on 18 April 2019 (CRIS Registration Number KCT0003808) and was conducted between February 2019 and May 2019.

### 2.2. Participants, Eligibility Criteria, and Recruitment

The inclusion criteria for our study were: (1) subjects who voluntarily agreed to participate and signed informed consent, and (2) healthy volunteers between the ages of 18 and 65 years old (including patients with controlled hypertension or diabetes mellitus). The exclusion criteria were as follows: (1) smokers; (2) alcohol consumption greater than 4 regular (250 cc) cups of beer, or 5 small (50 cc) glasses of Korean distilled spirits per day for men, and 2 regular (250 cc) cups of beer, or 2.5 small (50 cc) glasses of Korean distilled spirit per day for women, or drinking for more than two times per week; (3) pregnant women; (4) uncontrolled diabetes mellitus; (5) uncontrolled high blood pressure; (6) subjects with implanted cardiac pacemaker; (7) subjects taking antioxidant supplements, such as vitamin C, vitamin E, flavonoids, bilberry, CoQ19, glutathione, omega-3, Mg, Mn, and Zn (2 weeks of washout period was required before enrollment). Subjects were recruited through an announcement posted on a bulletin board inside Bundang Jesaeng Hospital in Korea between 30 January 2019 and 19 February 2019.

### 2.3. Study Setting and Interventions

This study was conducted at Bundang Jesaeng Hospital in Seongnam, South Korea, from February 2019 to May 2019. Five ERW-producing apparatus sets (AML 3010S, Alkamedi Co. Ltd., Anyang, Korea) were installed at five different locations throughout the hospital (OASIS-O area, OASIS-A area, OASIS-S1 area, OASIS-S2 area, and OASIS-I area). Each installed device consisted of two units. The first was a carbon filter unit that purified tap water for production of MW, whereas the second was an electrolysis unit consisting of five platinum (Pt)-coated titanium (Ti) electrode plates separated by semipermeable membranes for producing ERW [17,22]. Purified MW flows into the electrolysis unit, where it becomes electrolyzed while passing through the gap between the Pt-Ti electrodes [22]. ERW containing hydrogen (H_2_) gas and hydroxide ions (OH^−^) are produced at the cathode [12], whereas electrolyzed oxidized water containing oxygen (O_2_) gas and hydrogen ions (H^+^) are produced at the anode [12] by electrolysis of water. ERW produced by electrolysis devices generally exhibits high pH, low ORP, and high dissolved hydrogen [12,13,22]. In this study, the pH, ORP, dissolved hydrogen concentration, and electric conductivity of the ERW-producing apparatus were 9.39 ± 7.2, −111.36 ± 7.39 mV, 586.8 ± 20.5 μg/L, and 273.6 ± 0.52 µS/cm, respectively, whereas those for the MW-producing apparatus were 7.2 ± 0.1, 304.00 ± 6.74 mV, 0.00 ± 0.00 μg/L, and 273.5 ± 0.53 µS/cm, respectively. Additionally, the source tap water used in the study was distributed by Bokjeong Water Purification Plant, Seongnam, South Korea and had been demonstrated to be suitable for drinking based on the quality analysis conducted by the engineer of the department of Clean Water Management Office in Seongnam City Hall. Analysis of the tap water showed a pH of 7.5, residual chlorine level of 0.12 mg/L, turbidity of 0.06 NTU, Cu level of 0.017 mg/L, Fe level of < 0.3 mg/L, and nondetectable levels of Zn and Mn. However, the limited elemental profile analysis performed for both ERW and MW might have hindered a further thorough chemical comparison of these waters in the current study.

Sixty-five participants were allocated into two groups; of these, 61 received intervention (32 for ERW and 29 for MW) while the remaining four voluntarily withdrew prior to commencement of the study. Participants were supplied with bottles of the same shape and size. However, bottles were of two different colors (blue or green), were able to hold 750 cc of water, and participants were instructed to drink 1.5 L/day of water from the devices allocated to their group for eight weeks.

### 2.4. Outcome Variables

The primary outcome of our study was to evaluate oxidative stress biomarkers including measurement of serum biological antioxidant potential (BAP), diacron-reactive oxygen metabolites (d-ROMs), oxidized low-density lipoprotein (oxLDL), superoxide dismutase (SOD), and catalase (CAT) levels; urine malondialdehyde (MDA) and 8-hydroxydeoxyguanosine (8-OHdG) levels; and plasma glutathione peroxidase (GPx) levels. OxLDL, a parameter of protein oxidation, was added after the study commenced to estimate the oxidative stress in more diverse aspects. The secondary outcome was to evaluate health-related indices, including the biochemistry profile (aspartate aminotransferase (AST), alanine aminotransferase (ALT), low-density lipoprotein (LDL) cholesterol, triglyceride, glucose, insulin, homeostatic model assessment for insulin resistance (HOMA-IR), cortisol, total bilirubin, gamma-glutamyltransferase (GGT), uric acid, lactic acid, alkaline phosphatase (ALP), serum calcium, urine calcium), natural killer (NK) cell activity, advanced glycation end product (AGE) of skin, fat mass (total, visceral, and subcutaneous), cardio-ankle vascular index (CAVI), heart rate variability (HRV), whole-body phase angle (PhA), as well as implementation of questionnaires to assess stress (Brief Encounter Psychosocial Instrument - Korean version (BEPSI-K)), fatigue (Brief Fatigue Inventory (BFI) and Fatigue Severity Scale (FSS)), and Health-Related Quality of Life (HRQoL) (36-Item Short Form Survey (SF-36)). Primary and secondary outcome variables were assessed at baseline as well as after 4 weeks and 8 weeks of intervention.

### 2.5. Sample Size

The sample size was determined using the G Power 3.1 program [44]. Considering that few randomized controlled trials (RCT) have been performed to offer appropriate effect size for similar primary outcomes, Cohen’s convention for effect size (Cohen 1977) was applied, assuming detection of a medium effect (f = 0.25). Further, results from the Priori power analysis conducted to test the within-between interactions using repeated measured ANOVA with a medium effect size (f = 0.25), and an alpha of 0.05, determined that a total sample size of 42 participants with two equal-sized groups (*n* = 21) was required to achieve a power of 0.95 [44]. However, considering the potential dropout rate of 35%, the adjusted sample size (*N*) was 65 and denoted as Equation (1):

(1) $$N=\frac{n}{\left(1-d\right)}$$

where *N* = adjusted sample size, *n* = sample size required as per G power, and *d* = dropout rate [45,46].

### 2.6. Randomization, Allocation, and Blinding

Randomization was conducted by an independent investigator not otherwise involved in the clinical trial using the R package 3.5.2. (2018, The R Foundation for Statistical Computing) [47]. Accordingly, a 1:1 allocation to the green or blue group was accomplished using permuted block randomization with random block sizes of 4, 6, and 8. The allocation sequence was concealed in an opaque, sealed envelope, and provided to the research manager. The envelope was concealed until all enrolled subjects had provided informed consent and baseline assessments were completed. The research manager opened the envelope and assigned participants to the two groups according to the allocation sequence, while bottles of two different colors (blue or green) for drinking water were distributed to all participants.

The pH of the water in each electrolysis apparatus (ten apparatuses, five for ERW group, and five for MW group) was defined outside the hospital, before installation. Until the analysis of data, the pH of the water in each apparatus was blinded to investigators, participants, and the research manager by turning off the light of the LCD monitor on the surface of the apparatus, which otherwise showed the pH of the water in real-time. Meanwhile, a photo of each bottle (blue or green) pertaining to ERW or MW was attached on the surface of each apparatus by the engineer of the company who installed the apparatus. The bottle color for either the ERW or MW group was blinded to investigators, participants, and the research manager until the completion and analysis of the study. The participants assigned to the two groups were instructed to drink water from the apparatus on which the photo of the bottle of the same color with their group name was attached.

### 2.7. Measurement of Outcome Variables

After overnight fasting for 12 h, blood was collected from a vein in the cubital fossa using a BD vacutainer system (Becton Dickenson, Plymouth, UK) into 1 cc Promoca™ (for the activity of NK cells), 3 cc heparin (for lactic acid), 3 cc EDTA (for GPx and complete blood count (CBC) with differentials), and 8.5 cc SST (for other parameters) tubes. Urine samples were collected into 10 cc simple urine tubes (for urine Ca, thiobarbituric acid reactive substances (TBARS), and 8-OHdG).

Blood in SST tubes was allowed to clot at 25 °C for approximately 15 min and then centrifuged (Kubota 4000, Kubota corp., Tokyo, Japan) at 1710× *g* for 10 min. Tubes containing EDTA as anticoagulant were centrifuged at 430× *g* for 10 min to obtain plasma. A portion of the serum and plasma sample supernatants were pipetted into microtubes and stored at −80 °C until analysis. Urine samples were processed in the same way if immediate analysis was not available. Serum SOD, CAT, oxLDL, plasma GPx, urine TBARS, and 8-OHdG were analyzed at GC Labs (Yongin, South Korea), while NK cells activity was analyzed at ATGEN (Seongnam, South Korea). All other samples were analyzed at the Department of Laboratory Medicine, Bundang Jesaeng Hospital (Seongnam, South Korea).

#### 2.7.1. Biomarkers of Oxidative Stress

The d-ROMs and BAP test were performed according to the manufacturer’s instructions (Wismerll, Tokyo, Japan). The d-ROMs test evaluates the status of oxidative stress by determining hydroperoxides (ROOH) and diverse organic compounds (lipids, proteins, nucleic acids, etc.) levels [48] through detection of chromogenic substrate (N,N-diethyl-para-phenylenediamine) oxidation by radicals converted from hydroperoxides [49,50]. The reaction is photometrically monitored at 505 nm, and results are expressed as Carratelli Units (U.CARR), where 1 U.CARR corresponds to 0.8 mg/L H_2_O_2_. The standard reference level for d-ROMs was 250-300 U.CARR [51,52]. The BAP test provides a global measurement of many antioxidants, including uric acid, ascorbic acid, and bilirubin [53,54], based on the ability of a blood sample to reduce ferric ions to ferrous ions [53,54,55]. Urine MDA was measured using an enzyme-linked immunosorbent assay (ELISA) and the OxiSelect™ TBARS assay kit (Cell Biolabs, San Diego, CA, USA) according to the manufacturer’s instructions. The TBARS has been used as a tool for direct quantitative measurement of MDA [56]. Urine 8-OHdG was assessed by competitive ELISA using the “8-OHdG check” kit (JaICA, Fukuroi City, Japan) based on a monoclonal antibody specific for quantification of 8-OHdG [57,58]. Oxidized low-density lipoprotein (oxLDL) was determined using the Mercodia oxLDL ELISA kit (Mercodia, Uppsala, Sweden) according to manufacturer’s instruction [58]. All enzymatic activities were evaluated using assay kits (Cayman Chemicals, Ann Arbor, MI, USA) following the manufacturer’s instructions [59,60]. The absorbance of MDA, 8-OHdG, oxLDL, and GPx were measured colorimetrically at 532 nm, 450 nm, 450 nm, and 340 nm respectively, using an ELISA microplate reader (VersaMax, Molecular Devices, San Jose, CA, USA) [56,57,58,59,60].

#### 2.7.2. Biochemistry Parameters

The CBC was measured with an Advia 2120 (Siemens Healthcare Diagnostics, Erlangen, Germany), while the neutrophil to lymphocyte ratio (NLR) was calculated by dividing the absolute neutrophil count by the absolute lymphocyte count [61]. The levels of HbA1c were measured using an HLC-723^®^ G11 glycohemoglobin (HbA1c) automated analyzer (Tosho Co., Ltd., Tokyo, Japan) employing high-performance liquid chromatography. The levels of insulin and cortisol were measured using electro-chemiluminescence immunoassays (ECLIA) in a Cobas 8000-E602 module biochemistry analyzer (Roche Diagnostics, Basel, Switzerland), according to the standard operating procedures recommended by the manufacturer. We calculated HOMA-IR using the standard formula based on fasting glucose and insulin [54], according to Equation (2) [62]:

(2) $$HOMA-IR=\frac{\left[fasting\;glucose\right]\left(\frac{mg}{dL}\right)\left[fasting\;insulin\right]\left(µ\frac{g}{mL}\right)}{405}$$

Other biochemistry parameters were measured using the TBA2000FR automated chemistry analyzer (Toshiba, Tokyo, Japan) according to standard methods.

#### 2.7.3. Activity of NK Cells

The activity of natural killer cells was measured using the NK Vue™ kit (ATgen, Sungnam, Korea) according to the manufacturer’s instructions. This kit uses an immunomodulatory cytokine (Promoca) to stimulate NK cells in whole blood. The level of secreted interferon (IFN) is then determined using ELISA [63]. Absorbance was measured at 450 nm using a microplate reader (SpectraMax 190, Molecular Devices, LLC., San Jose, CA, USA).

#### 2.7.4. Advanced Glycation End Products (AGEs)

The AGEs were determined using an AGE Reader (DiagnOptics, Groningen, Netherlands), as previously described. The device illuminates a skin surface [64] using an excitation light source with a wavelength between 300 nm and 420 nm. Skin emits light at a wavelength range of 420–600 nm. Skin autofluorescence was then measured by calculating the ratio of the average reflected light intensity to the average emitted light intensity multiplied by 100 and was expressed in arbitrary units (AU) [65,66].

#### 2.7.5. Cardio-Ankle Vascular Index (CAVI)

The CAVI, used for assessment of arterial stiffness [67], was measured using the VASERA VS-1000 automatic pulse wave analyzer (Fukuda Denshi Co. Ltd., Tokyo, Japan) as previously described [62,68]. The arterial pressure waveforms of the brachial and ankle arteries, phonocardiography, and electrography were measured, and CAVI was automatically calculated by the analyzer [69,70,71].

#### 2.7.6. Heart Rate Variability (HRV)

Parameters of HRV, including the time and frequency domains monitoring the balance and activity of the autonomic nervous system, were analyzed using the SA-6000 heart rate variability analysis system (Medicore Co.,Ltd., Seoul, Korea) according to the manufacturer’s instructions [72,73]. The time domain parameters analyzed were the mean heart rate, standard deviation of the N-N interval (SDNN), square root of the mean squared differences of successive N-N intervals (RMSSD), and physical stress index (PSI), whereas the frequency domain parameters analyzed were the total (TP), low frequency (LF), and high frequency (HF) power. The ratio of the low frequency to high frequency (LF/HF ratio) was then calculated [72,73].

#### 2.7.7. PhA and Body Composition

PhA was measured using the InBody S10 multifrequency bioelectrical impedance analyzer (InBody, Seoul, Korea) [74]. The extracellular water (ECW), total body water (TBW), reactance (*Xc*), and impedance (*Z*) of right arm (*RA*), right leg (*RL*), left arm (LA), left leg (LL), and trunk were recorded [75]. PhA was calculated from reactance (*Xc*) and impedance (*Z*) (measured at 50 kHz) using Equation (3):

(3) $$PhA\;\left({}^{\circ}\right)=arcsin\left(\frac{Xc}{Z}\right)\left(\frac{180}{\pi }\right)$$

Total PhA was calculated according to Equation (4) measured at 50 kHz:

(4) $$Total\;PhA\;\left({}^{\circ}\right)=arcsin\;\left(\frac{sum\;of\;Xc\;of\;\left(RA+RL+TR\right)}{sum\;of\;R\;of\;\left(RA+RL+TR\right)}\right)$$

Overall body composition was measured using the X-SCAN PLUS 950 body composition analyzer (Selvas Healthcare, Daejun, Korea) in which body weight, height, body mass index (BMI), fat mass, visceral fat mass, and subcutaneous fat mass were measured.

#### 2.7.8. Blood Pressure and Pulse Rate

Blood pressure and pulse rate were measured using the BPBIO320 single-cuff oscillometer blood pressure (BP) monitor (Inbody, Seoul, Korea).

#### 2.7.9. Questionnaires to Evaluate Stress, Fatigue, and HRQoL

The BEPSI-K [76,77] was used to measure stress levels. Concomitantly, perceived fatigue was assessed using the validated BFI [78] and FSS [79]. The HRQoL was measured using the SF-36 questionnaire [80].

### 2.8. Data Analysis

Data were analyzed using the IBM SPSS Statistics for Windows Software (Version 22.0, Armonk, NY, USA). The values presented in the text were expressed as means (M) ± standard deviation (SD). The normality of the distribution of variables was evaluated using the Shapiro-Wilk test. When comparing the continuous variables between the ERW and MW groups at baseline, the Student’s *t*-test or the Mann-Whitney U test was used. For categorical response variables, the differences between the two groups were assessed using the Pearson’s Chi-squared test, Fisher’s exact test, or linear-by-linear association. To determine differences between groups over time, analysis of variance with repeated measures with the post hoc Bonferroni adjustment for multiple comparisons was performed. Results were considered to be significant at *p* < 0.05.

## 3. Results

**Figure 1:**
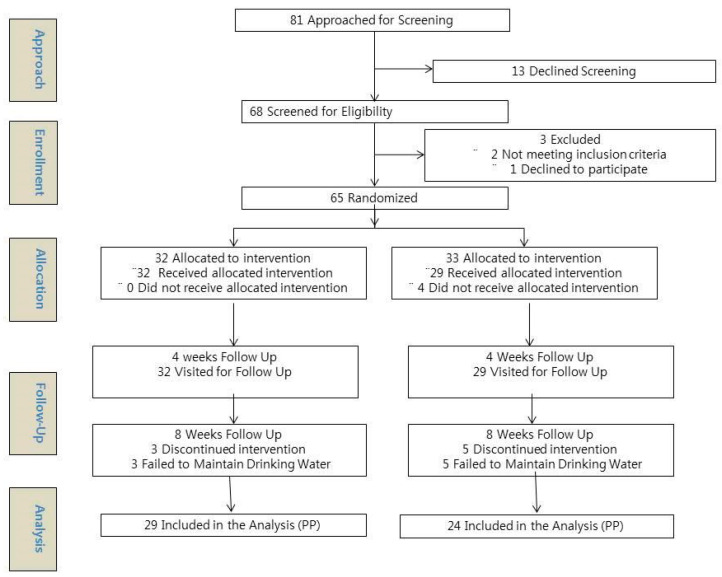
A Consolidated Standards of Reporting Trials (CONSORT) flow diagram.(corrected in the separate files)

Initially, 81 subjects were approached for screening. Of these, 13 declined while 68 consented to undergo screening, of which two subjects did not meet the inclusion criteria and one refused to participate in the study. Accordingly, 65 subjects, meeting the inclusion and exclusion criteria, and who had provided written informed consent, were enrolled in the study and randomly assigned into the ERW (*n* = 32) or MW group (*n* = 33). A total of 61 subjects received the allocated intervention and underwent 4-week follow-up evaluation, whereas three and five subjects in the ERW and MW group, respectively, discontinued intervention before 8-week follow-up, owing to their inability to continue drinking the recommended volume of water. After the scheduled follow-up, 53 subjects, 29 in the ERW and 24 in the MW group, completed the study and were assessed for primary and secondary outcomes (per-protocol (PP) analysis) (Figure 1).

### 3.1. Baseline Characteristics of Study Participants

Baseline demographic and clinical characteristics of the study participants are presented in Table 1. No significant differences were observed between the two groups, except for d-ROMs, the *p*-value of which was only slightly below 0.05 (*p* = 0.046) (Table 1).

### 3.2. Effect of Treatment on the Primary Outcome Variables: Biomarkers of Oxidative Stress

Data were analyzed via repeated measure ANOVA using a within-subject factor of time, and a between-subject factor of group. The ERW group displayed a reduction in TBARS, 8-OHdG, and d-ROMs levels (mean ± SD) and an increase in BAP and GPx levels (mean ± SD) from baseline to 4 and 8 weeks follow-up (Table 2 and Supplementary Table S2).

The effect of time showed a significant difference in mean d-ROMs levels at different time points, F(1.68, 85.65) = 6.61, *p* = 0.004, partial η^2^ = 0.115; and the main effect of the group displayed a significant difference in the mean d-ROMs levels between the two groups, F(1, 51) = 8.04, *p* = 0.007, partial η^2^ = 0.136. There was a significant interaction observed between the intervention and time on the d-ROMs levels, F(1.68, 85.65) = 3.44, *p* = 0.044, partial η^2^ = 0.063 (Table 2 and Table S2). Follow-up assessments were carried out to determine the difference of d-ROMs levels between groups at each time point and the effect of time on d-ROMs levels in each group. A significant difference was observed in the serum d-ROMs levels between the two groups at 8 weeks intervention, F(1, 51) = 15.87, *p* < 0.001, partial η^2^ = 0.237; and serum d-ROMs levels were significantly lower in the ERW compared to those in the MW group (mean difference (M_diff_) = 61.66, standard error (SE) = 15.48 U.CARR, *p* < 0.001). There was also a significant effect of time on d-ROMs levels in the MW group, F(1.59, 36.58) = 6.09, *p* = 0.005, partial η^2^ = 0.209. Furthermore, d-ROMs levels were significantly lower at 4 weeks compared to those at baseline (M_diff_ = 41.04, SE = 14.06 U.CARR, *p* = 0.023).

Mean BAP levels significantly differed between time points, F(2,102) = 258.47, *p* < 0.001, partial η^2^ = 0.835. Regarding the effect of the group, no significant difference was observed in the mean BAP levels between the two groups, F(1,51) = 0.03, *p* = 0.875, partial η^2^ < 0.001. However, a significant interaction was observed between the group and time on BAP levels, F(2,102) = 3.20, *p* = 0.045, partial η^2^ = 0.059 (Table 2 and Table S2). Follow-up assessments for the effect of each intervention revealed slightly, yet not significantly, higher BAP levels in the ERW compared to the MW group at 4 weeks, F(1,51) =1.64, *p* = 0.206, partial η^2^ = 0.031. There was a significant effect of time on BAP levels in the ERW group, F(2,56) = 179.46, *p* < 0.001, partial η^2^ = 0.865; as well as in the MW group, F(2,46) = 97.36, *p* < 0.001, partial η^2^ = 0.809. In the ERW group, BAP levels were significantly higher at 4 weeks than those at baseline (M_diff_ = 558.52, SE = 32.40 umol/L, *p* < 0.001) and at 8 weeks than those at baseline (M_diff_ = 576.45, SE = 38.73 umol/L, *p* < 0.001). Meanwhile, in the MW group, BAP levels were significantly higher at 4 weeks (M_diff_ = 489.04, SE = 53.66 umol/L, *p* < 0.001) and 8 weeks (M_diff_ = 654.71, SE = 51.49 umol/L, *p* < 0.001) than those at baseline and at 8 weeks than those at 4 weeks (M_diff_ = 165.67, SE = 40.14 umol/L, *p* = 0.001).

Mean TBARS, 8-OHdG, and GPx levels significantly differed between time points (TBARS: F(2,102) = 6.23, *p* = 0.003, partial η^2^ = 0.109; 8-OHdG: F(2,102) = 3.52, *p* = 0.033, partial η^2^ = 0.065; GPx: F(2,102) = 27.07, *p* < 0.001, partial η^2^ = 0.347); however, these levels were not significantly different between the two groups (TBARS: F(1,51) = 0.06, *p* = 0.809, partial η^2^ = 0.001; 8-OHdG: F(1,51) = 0.29, *p* = 0.594, partial η^2^ = 0.006; GPx: F(1,51) = 0.22, *p* = 0.639, partial η^2^ = 0.004). The interaction between the group and time did not significantly influence TBARS (F(2,102) = 2.45, *p* = 0.091, partial η^2^ = 0.046), 8-OHdG (F(2,102) = 2.02, *p* = 0.138, partial η^2^ = 0.038), and GPx (F(2,102) = 0.89, *p* = 0.412, partial η^2^ = 0.017) levels. Mean oxLDL levels did not significantly differ at different time points (F(2,102) = 1.11, *p* = 0.332, partial η^2^ = 0.021) or between the two groups (F(1,51) = 0.17, *p* = 0.679, partial η^2^ = 0.003). The interaction between the group and time did not display a significant effect on mean oxLDL levels (F(2,102) = 0.63, *p* = 0.534, partial η^2^ = 0.012) (Table 2 and Table S2).

### 3.3. Effect of Treatment on the Secondary Outcome Variables: Biochemistry Parameters

Mean NK cell activity significantly differed between time points, F(2,102) = 20.94, *p* < 0.001, partial η^2^ = 0.291. In contrast, the intervention did not exert a significant effect on mean NK cell activity between the two groups, F(1,51) = 0.40, *p* = 0.528, partial η^2^ = 0.008. The interaction between the group and time did not significantly influence mean NK cell activity, F(2,102) = 1.26, *p* = 0.289, partial η^2^ = 0.024 (Table 3 and Table S3).

In both intervention groups, AGE, insulin, HOMA-IR, and cortisol levels were lower at 4 and 8 weeks than those at baseline. Regarding the effect of time, a significant difference was observed in the mean AGE, F(2,102) = 20.94, *p* < 0.001, partial η2 = 0.291 at different time points. Regarding variables other than AGE, the effect of time did not display a significant difference. Similarly, no significant difference was observed in the effect of the intervention itself. Furthermore, the group and amount of time did not have an interactive effect on AGE or other biochemical profiles (Table 3 and Table S3).

### 3.4. Effect of Treatment on the Secondary Outcome Variables: Body Composition, CAVI, HRV, and PhA

Significant interactions between the group and time were observed in total fat mass, F(1.80,91.60) = 3.43, *p* = 0.041, partial η^2^ = 0.063, visceral fat mass, F(2,102) = 3.76, *p* = 0.027, partial η^2^ = 0.069, and subcutaneous fat mass, F(1.78,90.50) = 3.29, *p* = 0.047, partial η^2^ = 0.061 (Table 4 and Table S4). Regarding the effect of time, significant differences were observed in total fat mass, F(1.80,91.60) = 10.12, *p* < 0.001, partial η^2^ = 0.166, visceral fat mass F(2,102) = 9.96, *p* < 0.001, partial η^2^ = 0.163, and subcutaneous fat mass, F(1.78,90.50) = 9.81, *p* < 0.001, partial η^2^ = 0.161, at different time points (Table 4 and Table S4). Follow-up assessments were performed for fat mass to determine the differences between the groups at each time point and the effect of time in each group. However, the intervention itself at 4 and 8 weeks did not significantly influence total, visceral, or subcutaneous fat mass. Alternatively, the effect of time significantly influenced total fat mass in the MW group, F(2, 46) = 7.47, *p* = 0.002, partial η^2^ = 0.245, but not in the ERW group, F(1.49,41.74) = 2.40, *p* = 0.116, partial η^2^ = 0.079. In the MW group, total fat mass was significantly lower at 8 weeks than at baseline (M_diff_ = 1.208, SE = 0.317 kg, *p* = 0.003). The effect of time also significantly influenced the visceral fat mass both in the ERW, F(2,56) = 3.80, *p* = 0.028, partial η^2^ = 0.120, and MW, F(2,46) = 7.06, *p* = 0.002, partial η^2^ = 0.235 groups, being significantly lower at 8 weeks than at 4 weeks in the ERW group (M_diff_ = 0.083, SE = 0.028 kg, *p* = 0.019), and significantly lower at 8 weeks than at baseline in the MW group (M_diff_ = 0.212, SE = 0.058 kg, *p* = 0.004). However, the effect of time significantly influenced subcutaneous fat mass in the MW group, F(2,46) = 7.32, *p* = 0.002, partial η^2^ = 0.241, but not in the ERW group, F(1.44,40.21) = 2.11, *p* = 0.147, partial η^2^ = 0.070; in the former, subcutaneous fat mass was significantly lower at 8 weeks than at baseline (M_diff_ = 0.996, SE = 0.263 kg, *p* = 0.003).

Both the right and left mean CAVI decreased with time in the ERW group, while neither displayed a significant difference in the primary effect for either time point, F(1.45,73.80) = 0.36, *p* = 0.627, partial η^2^ = 0.007 (Rt), F(1.44,73.30) = 0.38, *p* = 0.444, partial η^2^ = 0.014 (Lt); or group, F(1,51) = 0.11, *p* = 0.738, partial η^2^ = 0.002 (Rt), F(1,51) = 0.32, *p* = 0.577, partial η^2^ = 0.006 (Lt). Similarly, no significant difference was observed in the interaction between the group and time, F(1.45,73.80) = 0.38, *p* = 0.615 partial η^2^ = 0.007 (Rt), F(1.44,73.30) = 0.29, *p* = 0.677, partial η^2^ = 0.006 (Lt) (Table 4 and Table S4).

Regarding the time domain parameters of HRV, PSI decreased with time, whereas TP increased through the study period in the ERW group. However, none of the HRV parameters differed significantly in terms of the effect of time, the effect of the group, or the interaction between the group and time (Table 4 and Table S4).

All parameters of PhA were not significantly different in the effect of the intervention, and the interaction between the group and time (Table 4 and Table S4).

### 3.5. Effect of Treatment on the Secondary Outcome Variables, BEPSI-K, BFI, FSS, and SF-36 Score

Herein, although the BEPSI-K score tended to decrease with time in the ERW group, the interaction between group and time did not significantly influence the BEPSI-K score (F(2,102) = 0.68, *p* = 0.511, partial η^2^ = 0.013); similarly, the mean BEPSI-K score did not significantly differ between the two groups (F(1,51) = 0.01, *p* = 0.907, partial η^2^ < 0.001, or at different time points, F(2, 102) = 0.60, *p* = 0.553, partial η^2^ = 0.012) (Table 5 and Table S5).

The BFI global score, BFI severity score, and BFI interference score were significantly influenced by the treatment duration, [F(2,102) = 4.28, *p* = 0.016, partial η^2^ = 0.077], [F(2,102) = 3.11, *p* = 0.049, partial η^2^ = 0.057], and [F(2,102) = 3.72, *p* = 0.028, partial η^2^ = 0.068], respectively; however, they were not significantly influenced by the group ([F(1,51) < 0.01, *p* = 0.964, partial η^2^ < 0.001], [F(1,51) = 0.33, *p* = 0.566, partial η^2^ = 0.007], and [F(1,51) = 0.04, *p* = 0.845, partial η^2^ = 0.001], respectively) or by the interaction between the group and time ([F(2,102) = 0.11, *p* = 0.901, partial η^2^ = 0.002], [F(2,102) = 0.05, *p* = 0.956, partial η^2^ = 0.001], and [F(2,102) = 0.11, *p* = 0.895, partial η^2^ = 0.002], respectively) (Table 5 and Table S5).

The FSS score was lower at 4 and 8 weeks than at baseline in both groups; however, the effect of the groups (F(1,51) = 0.01, *p* = 0.928, partial η^2^ < 0.001), the effect of time (F(1.74,88.48) = 1.83, *p* = 0.172, partial η^2^ = 0.035), and the interaction between the group and time (F(1.74,88.48) = 0.68, *p* = 0.489, partial η^2^ = 0.013) did not significantly influence the FSS score (Table 5 and Table S5).

The group, duration of treatment, and interaction between the group and time did not significantly influence the scores of every item of SF-36 (Table 5 and Table S5).

## 4. Discussion

This study was the first randomized, double-blind, placebo-controlled trial to assess the outcomes of specific lifestyle modification, namely drinking ERW for eight weeks at the workplace, in healthy adults. The assessment was based on a comprehensive evaluation of biomarkers of oxidative stress, encompassing reactive oxygen metabolites, total antioxidant potential, oxidative stress-induced damaged macromolecules (lipid, DNA, and protein), and antioxidant enzymes as primary outcomes, as well as various health-related indices of altered medical conditions resulting from oxidative stress as secondary outcomes. Diverse biochemical parameters and fat mass were measured to assess the effect of ERW on glucose and lipid metabolism as well as on body composition. To assess effects on arterial stiffness, the cardio-ankle vascular indices were measured. HRV was measured to evaluate the change in cardiac autonomic function after drinking ERW, and phase angle was measured as an index of cellular health. The effects of drinking ERW on stress, fatigue, and quality of life were assessed by using BEPSI-K, BFI and FSS, and SF-36 questionnaires.

The majority of healthy adults spend most of their waking hours at work, so the workplace appears to be the ideal place to adopt a healthy lifestyle. Hence, health promotion programs adopting lifestyle modifications at the workplace are being implemented in several countries. There have been several randomized clinical trials assessing the effects of healthy behaviors at the workplace [39,40,41,42], with some meeting their primary endpoints, whereas others not meeting their primary endpoints. Meanwhile, such trials have raised questions regarding sustainability, affordability, and cost-effectiveness [38,43]. Successful and sustainable programs based on lifestyle modifications must be accessible [81], affordable, effective, and equitable to all individuals without discrimination or disparities [43,82,83].

Although there are diverse biomarkers available for the estimation of the status of oxidative stress, there continues to be a lack of consensus on which biomarkers are best suited for measuring redox states. Hence, usually, several biomarkers are measured together, including free radicals, ROS, oxidative stress-induced damaged macromolecules, antioxidant capacity, and antioxidant enzymes for antioxidant potential. Although it would be ideal to directly measure reactive species [84], this approach was excluded from our study because of their high reactivity and short half-life [84].

The BAP and d-ROMs tests have been widely used as biomarkers measuring antioxidant activity and oxidative stress as a whole [52,55,84]. In this study, both biomarkers demonstrated a significant association between the group and timely intervention, indicating that eight weeks of consuming ERW may prove effective in both reducing oxidative stress and increasing antioxidant potential compared to drinking MW. Electrolysis of water produces hydrogen molecules and hydrogen atoms (active hydrogen) in the vicinity of the platinum plate at the cathode [12], both of which have been shown to contribute to reduced oxidative stress by scavenging ROS [12]. Additionally, platinum nanoparticles (PtNPs) and PtNP-hybrids formed near the cathode seem to scavenge ROS, including superoxide radicals, hydroxyl radicals, and hydrogen peroxide [12]. The MDA, OHdG, and oxLDL biomarkers were measured to evaluate the oxidation of proteins, DNA, and lipids by free radicals, respectively. Regarding MDA and OHdG, treatment revealed a significant difference in the mean concentration of MDA and OHdG at different time points but did not exhibit significant interaction between the group and timely intervention. The concentration of MDA and 8-OHdG in this study was measured using spot urine. However, there has been a controversy over whether to normalize biomarkers in urine with the concentration of urinary creatinine (μCr) since the rate of excretion of urinary creatinine may change according to age, sex, exercise, diet, stress, muscle mass, and stress [85]. Approximately 20% of the subjects were exercising at various intensities during the study period, so we chose not to perform normalization in this study, considering the potential effect on the concentration of urine creatinine.

It is known that ROS may change the surface charge of NK cells to negative, thereby disturbing the attachment of NK cells to target cancer cells, which are usually anionic [86]. pH-centered cancer care is attracting more and more attention in the field of oncology [87], and numerous studies have been attempted to modify the alkaline intracellular and acidic extracellular microenvironment of cancer cells [87,88]. The activity of NK cells showed a great increase from baseline (949.0 ± 810.68) to 4 weeks (1943.5 ± 1109.2) and 8 weeks (1814.2 ± 1091.8) in both the ERW and MW groups in this study. We may, therefore, cautiously postulate from the perspective of nutritional immunology that drinking ERW may be a potential candidate for cancer prevention [89,90,91]. Most of the tested biochemistry parameters did not demonstrate a statistical difference in the interaction between time and group even though several parameters related to glucose metabolism (AGE, insulin, HOMA-IR, and cortisol) showed a tendency to decrease at 4 and 8 weeks compared to those at baseline. Besides, the ion concentrations of ERW and MW are possibly different, and these differences might have also influenced the observed differences in biochemical parameters before and after drinking ERW and MW. The lack of ion concentration measurement may be considered as a limitation of this study.

Although our results showed a decreased tendency in BEPSI-K, BFI, and FSS scores at 4 and 8 weeks of intervention compared to those at baseline, the interaction between the group and time did not reach statistical significance. Furthermore, each SF-36 item did not exhibit significant differences over time between the two groups.

Notably, most biomarkers of oxidative stress and health-related indices showed less improvement during the last 4 weeks compared to the first 4 weeks, and this effect may be attributed to the observed difference in the average intake of water between the two periods (1481 ± 78 vs. 1311 ± 201, *p* < 0.001). For instance, reduced intake of water due to busier schedules during the last 4 weeks might have affected the differences shown in outcome variables between the 4 and 8 weeks. There was one study related to the intake of blueberry, showing a blunted improvement of biomarkers of oxidative stress later in the intervention. One of the diverse interpretations suggested for this finding was that there might have been an acclimation leading to reduced oxidative damage over time [92]. More study subjects and more extended study periods are needed to verify the acclimation effect on oxidative biomarkers [92]. Hence, to more precisely characterize the clinical and statistical significance of the effect elicited by drinking ERW on various biochemical parameters, stress, fatigue, and quality of life in terms of lifestyle modification at the workplace, studies involving a larger sample size of participants over a more extended study period, with efforts to reduce the number of participant dropouts, are warranted.

In body composition analysis, total, visceral, and subcutaneous fat mass showed a more significant decrease over time in the ERW relative to the MW group. ERW might have directly induced lipolysis in adipocytes, downregulated the expression of transcription factors in the adipogenesis pathway, or reduced accumulation of lipids by affecting the expression of genes, such as fatty acid synthase (FAS), lipoprotein lipase (LPL), or hormone-sensitive lipase (HSL) during the differentiation of preadipocytes [93,94,95]. Additionally, ERW might have influenced the production of leptin or adiponectin [94,96], which would have accounted for the observed fat mass reduction. A colonization pattern of microbiota has been known to be one of the contributing factors of obesity, while a higher ratio of Firmicutes over Bacteroidetes has been demonstrated as a pattern commonly seen in obese people [97,98,99,100]. One study showed that anaerobes and aerobes grow at environments of different oxidation-reduction potential (ORP), thus drinking ERW might favor the growth of anaerobes [101].

On the other hand, the ORP is an important component showing the antioxidant potential of the reduced water. In a previous study comparing the ORP of reduced water with different pH, an ERW sample with a pH close to 10 showed considerably higher negative ORP value in vitro compared to ERW with a pH of 9.5 [102]. In future studies, it is recommended to compare ERW with different pH and ORP values to find out the best outcome in terms of antioxidant potential. Platinum coated on titanium electrode plates is already commonly used and known to be effective for electrolysis; further research on new materials, such as Nickel-Cobalt-Titanium alloy, that may increase ORP and the cost-effectiveness [103] will allow more individuals to benefit from drinking ERW.

## 5. Conclusions

This study was an RCT employed to determine the effects of ERW on biomarkers of oxidative stress and health-related indices in healthy adults. Drinking of ERW improved the major biomarkers of oxidative stress and body fat mass. Significant interactions were observed between the intervention and time on d-ROMs, BAP, and fat mass. Hence, drinking ERW on a daily basis may be effective in the reduction of oxidative stress and increase of oxidant potential, as well as in the decrease of total body, visceral, and subcutaneous fat mass. However, additional research participants with a more extended study period as well as analysis of the ionic concentrations of ERW and MW are needed in future studies to definitively support the adoption of drinking ERW at the workplace as an effective, equitable, accessible, sustainable, and innovatively simple lifestyle modification for health promotion.

## Figures and Tables

**Table 1:** Baseline characteristics of study participants.

Characteristic		ERW (*n* = 29)	MW (*n* = 24)	*p*-Value
**Demographic**	years			
Age		39.3 ± 8.9	41.0 ± 9.3	0.523 *
Sex	no. (%)			1.000 ^†^
Female		25 (86.2%)	21 (87.5%)	
Male		4 (13.8%)	3 (12.5%)	
Marital status	no. (%)			0.378 ^‡^
Married		18 (62.1%)	12 (50.0%)	
Single		11 (37.9%)	12 (50.0%)	
Educational level	no. (%)			0.701 ^§^
High school		3 (10.3%)	2 (8.3%)	
College/University		21 (72.4%)	17 (70.8%)	
Postgraduate		5 (17.2%)	5 (20.8%)	
Occupation	no. (%)			0.886 ^§^
Professionals		6 (20.7%)	5 (20.8%)	
White collar		20 (69.0%)	17 (70.8%)	
Blue collar		3 (10.3%)	2 (8.3%)	
**Clinical**				
d-ROMs	U.CARR	301.3 ± 68.6	347.3 ± 94.9	0.046 *
BAP	umol/L	2027.4 ± 226.9	2015.7 ± 274.8	0.734 ^‖^
TBARS(MDA)	μM	9.88 ± 4.67	8.90 ± 5.98	0.506 *
8-OHdG	ng/mL	14.33 ± 6.88	10.90 ± 6.42	0.069 *
oxLDL	U/L	52.43 ± 17.38	49.38 ± 13.61	0.520 ^‖^
GPx	nmol/min/mL	151.4 ± 31.1	140.1 ± 34.9	0.180 ^‖^
Body Mass Index	kg/m^2^	22.2 ± 3.1	23.0 ± 3.1	0.357 *
Blood pressure	mmHg			
Systolic		116.3 ± 10.6	116.3 ± 9.5	0.943 ^‖^
Diastolic		72.0 ± 11.6	72.4 ± 9.5	0.971 ^‖^
Glucose	mg/dL	92.8 ± 7.5	92.3 ± 6.9	0.805 *

Note: Continuous variables are represented as means ± standard deviations (SD) and compared by * T test or ^‖^ Mann-Whitney U Test. Categorical variables are given as numbers (no) with percentages (%) and compared by ^‡^ Pearson’s Chi-Squared Test, ^†^ Fisher’s Exact Test, or ^§^ Linear-by-Linear Association as appropriate. Abbreviations: ERW, electrolyzed-reduced water; MW, mineral water; d-ROMs, diacron-reactive oxygen metabolites; BAP, biological antioxidant potential; TBARS, thiobarbituric acid reactive substances; MDA, malondialdehyde; 8-OHdG, 8-hydroxy-2-desoxyguanosine; oxLDL, oxidized low-density lipoprotein; GPx, glutathione peroxidase.

**Table 2:** Effect of treatment on the primary outcome variables: biomarkers of oxidative stress.

	ERW (*n* = 29)	MW (*n* = 24)	*p*		
Outcome Variables	Means ±SD	Means ±SD	Time	Group	Time * Group
d-ROMs(U.CARR)					
Baseline	301.3 ± 68.6	347.3 ± 94.9			
4 weeks	286.7 ± 45.1	306.3 ± 54.8	0.004	0.007	0.044
8 weeks	288.0 ± 50.0	349.6 ± 62.7			
BAP (umol/L)					
Baseline	2027.4 ± 226.9	2015.7 ± 274.8			
4 weeks	2585.9 ± 258.8	2504.8 ± 187.8	0.000	0.875	0.045
8 weeks	2603.9 ± 255.9	2670.4 ± 180.2			
TBARS (MDA) (μM)					
Baseline	9.88 ± 4.67	8.90 ± 5.98			
4 weeks	6.84 ± 3.99	8.95 ± 6.14	0.003	0.809	0.091
8 weeks	6.97 ± 4.51	6.63 ± 4.47			
8-OHdG (ng/mL)					
Baseline	14.33 ± 6.88	10.90 ± 6.42			
4 weeks	13.87 ± 8.31	15.63 ± 11.18	0.033	0.594	0.138
8 weeks	11.87 ± 7.90	10.87 ± 7.00			
oxLDL (U/L)					
Baseline	52.43 ± 17.38	49.38 ± 13.61			
4 weeks	53.60 ± 15.32	52.00 ± 12.94	0.332	0.679	0.534
8 weeks	52.24 ± 15.97	52.1 ± 12.99			
GPx (nmol/min/mL)					
Baseline	151.4 ± 31.1	140.1 ± 34.9			
4 weeks	162.8 ± 36.5	161.7 ± 37.9	0.000	0.639	0.412
8 weeks	185.8 ± 30.9	189.6 ± 23.6			

Note: * Interaction between Time and the Group. Abbreviations: ANOVA, analysis of variance; d-ROMs, diacron-reactive oxygen metabolites; BAP, biological antioxidant potential; TBARS, thiobarbituric acid reactive substances; MDA, malondialdehyde; 8-OHdG, 8-hydroxy-2-desoxyguanosine; oxLDL, oxidized low-density lipoprotein; GPx, glutathione peroxidase.

**Table 3:** Effects of treatment on the secondary outcome variables: biochemistry parameters.

	ERW (*n* = 29)	MW (*n* = 24)	*p*		
Outcome Variables	Means ±SD	Means ±SD	Time	Group	Time * Group
NK Cell Activity(pg/mL)					
Baseline	949.0 ± 810.7	1263.4 ± 905.2			
4 weeks	1943.5 ± 1109.2	1829.0 ± 1136.7	0.000	0.528	0.289
8 weeks	1814.2 ± 1091.8	2042.0 ± 964.9			
Glucose (mg/dL)					
Baseline	92.8 ± 7.5	92.3 ± 6.9			
4 weeks	92.8 ± 8.2	92.1 ± 6.0	0.978	0.640	0.916
8 weeks	93.0 ± 5.9	91.8 ± 6.2			
HbA1c(mg/dL)					
Baseline	5.2 ± 0.3	5.3 ± 0.3			
4 weeks	5.3 ± 0.3	5.4 ± 0.2	0.000	0.228	0.971
8 weeks	5.3 ± 0.3	5.4 ± 0.3			
Insulin (uU/mL)					
Baseline	8.65 ± 3.78	8.21 ± 10.31			
4 weeks	6.66 ± 3.19	7.18 ± 4.76	0.091	0.729	0.401
8 weeks	7.15 ± 3.19	8.50 ± 6.78			
HOMA-IR					
Baseline	2.21 ± 0.93	1.95 ± 2.74			
4 weeks	1.55 ± 0.80	1.65 ± 1.15	0.112	0.739	0.575
8 weeks	1.68 ± 0.80	1.98 ± 1.61			
A.G.E. (AU)					
Baseline	2.05 ± 0.26	2.04 ± 0.27			
4 weeks	1.83 ± 0.32	1.82 ± 0.23	0.000	0.767	0.246
8 weeks	1.82 ± 0.34	1.91 ± 0.33			
Cortisol(ng/mL)					
Baseline	80.81 ± 35.53	81.78 ± 25.30			
4 weeks	76.95 ± 27.36	79.06 ± 41.63	0.743	0.443	0.308
8 weeks	72.63 ± 27.47	85.70 ± 26.27			
Lactic Acid(mmol/L)					
Baseline	1.76 ± 0.86	1.52 ± 0.55			
4 weeks	1.65 ± 0.65	1.58 ± 0.61	0.454	0.699	0.093
8 weeks	1.44 ± 0.45	1.61 ± 0.47			

Note: * Interaction between Time and the Group. Abbreviations: NK, natural killer; HbA1c, glycosylated hemoglobin; HOMA-IR, homeostatic model assessment for insulin resistance, A.G.E., advanced glycation end products; AU, arbitrary units.

**Table 4:** Effects of treatment on the secondary outcome variables: fat mass, CAVI, HRV, and PhA.

	ERW (*n* = 29)	MW (*n* = 24)	*p*		
Outcome Variables	Means ± SD	Means ± SD	Time	Group	Time * Group
Fat Mass-Total (kg)					
Baseline	16.21 ± 4.88	17.30 ± 5.03			
4 weeks	16.18 ± 5.00	16.55 ± 4.90	0.000	0.678	0.041
8 weeks	15.86 ± 4.86	16.10 ± 5.31			
Fat Mass-Visceral (kg)					
Baseline	1.86 ± 0.96	2.00 ± 0.95			
4 weeks	1.87 ± 1.00	1.87 ± 0.82	0.000	0.846	0.027
8 weeks	1.79 ± 0.94	1.80 ± 0.93			
Fat Mass-Subcutaneous (kg)					
Baseline	14.36 ± 3.96	15.30 ± 4.12			
4 weeks	14.30 ± 4.04	14.69 ± 4.11	0.000	0.644	0.047
8 weeks	14.07 ± 3.96	14.30 ± 4.41			
CAVI-Rt					
Baseline	6.44 ± 0.80	6.36 ± 1.06			
4 weeks	6.32 ± 0.80	6.36 ± 0.73	0.627	0.738	0.615
8 weeks	6.18 ± 0.69	6.36 ± 0.86			
CAVI-Lt					
Baseline	6.52 ± 0.77	6.48 ± 1.11			
4 weeks	6.38 ± 0.83	6.48 ± 0.76	0.444	0.577	0.677
8 weeks	6.23 ± 0.69	6.42 ± 0.81			
HRV-PSI					
Baseline	71.92 ± 81.46	52.85 ± 31.23			
4 weeks	71.54 ± 59.39	54.99 ± 33.62	0.825	0.182	0.765
8 weeks	63.09 ± 41.19	54.68 ± 38.06			
HRV-TP (ms^2^)					
Baseline	913.43 ± 613.60	1071.63 ± 876.97			
4 weeks	985.86 ± 1030.49	1041.21 ± 1223.80	0.502	0.795	0.851
8 weeks	1185.43 ± 1570.90	1115.73 ± 973.82			
HRV-LF/HF					
Baseline	1.52 ± 1.30	2.01 ± 2.14			
4 weeks	1.64 ± 1.41	1.64 ± 1.79	0.629	0.958	0.197
8 weeks	2.19 ± 2.43	1.64 ± 1.34			
Phase Angle (° )					
Baseline	5.89 ± 0.85	5.83 ± 0.85			
4 weeks	5.63 ± 0.75	5.67 ± 0.78	0.000	0.952	0.566
8 weeks	5.62 ± 0.67	5.60 ± 0.84			

Note: * Interaction between Time and the Group. Abbreviations: CAVI, cardio-ankle vascular index; HRV, heart rate variability; PSI, physical stress index; TP, total power; LF, low frequency; HF, high frequency.

**Table 5:** Effects of treatment on the secondary outcome variables: BEPSI-K, BFI, FSS, and SF-36.

	ERW (*n* = 29)	MW (*n* = 24)	*p*		
Outcome Variables	Mean ± SD	Mean ± SD	Time	Group	Time * Group
BEPSI-K					
Baseline	1.82 ± 0.65	1.73 ± 0.51			
4 weeks	1.71 ± 0.72	1.73 ± 0.51	0.553	0.907	0.511
8 weeks	1.76 ± 0.73	1.78 ± 0.56			
BFI					
BFI Global					
Baseline	4.14 ± 2.09	4.01 ± 2.27			
4 weeks	3.60 ± 1.98	3.56 ± 2.09	0.016	0.964	0.901
8 weeks	3.28 ± 2.29	3.38 ± 2.28			
BFI Severity					
Baseline	5.69 ± 2.06	5.36 ± 2.45			
4 weeks	5.49 ± 1.77	5.14 ± 2.28	0.049	0.566	0.956
8 weeks	4.90 ± 2.32	4.71 ± 2.40			
BFI Interference					
Baseline	3.36 ± 2.39	3.33 ± 2.38			
4 weeks	2.66 ± 2.37	2.76 ± 2.30	0.028	0.845	0.895
8 weeks	2.47 ± 2.46	2.72 ± 2.41			
FSS					
Baseline	3.58 ± 1.58	3.65 ± 1.68			
4 weeks	3.15 ± 1.53	3.40 ± 1.67	0.172	0.928	0.489
8 weeks	3.39 ± 1.81	3.17 ± 1.65			

Note: * Interaction between Time and the Group. Abbreviations: BEPSI-K, Brief Encounter Psychosocial Instrument - Korean version; BFI, brief fatigue inventory; FSS, fatigue severity scale; SF-36, 36-item short-form survey.

## Supplementary Materials

The following are available online at https://www.mdpi.com/2076-3921/9/7/564/s1, Table S1: Baseline Characteristics of the Study Participants, Table S2: Effect of Treatment on the Primary Outcome Variables: Biomarkers of Oxidative Stress, Table S3: Effects of Treatment on the Secondary Outcome Variables: Biochemistry Parameters, Table S4: Effects of Treatment on the Secondary Outcome Variables: CAVI, HRV, PhA, and Fat Mass, Table S5: Effects of Treatment on the Secondary Outcome Variables: BEPSI-K, BFI, FSS, and SF-36.
