# Supplementary material for: Outcomes Assessment of Sustainable and Innovatively Simple Lifestyle Modification at the Workplace-Drinking Electrolyzed-Reduced Water (OASIS-ERW): A Randomized, Double-Blind, Placebo-Controlled Trial
Source: Antioxidants (Basel). 2020 Jun 27;9(7):564. doi: 10.3390/antiox9070564 (PMC7402115; doi:10.3390/antiox9070564)
Supplement: Supplementary file 1 [file antioxidants-09-00564-s001.pdf]

Supplementary Materials (Table **S1-** Table **S5)**

Table S1: Baseline Characteristics of the Study Participants

Table S2: Effect of Treatment on the Primary Outcome Variables: Biomarkers of Oxidative Stress

Table S3: Effects of Treatment on the Secondary Outcome Variables: Biochemistry Parameters

Table S4: Effects of Treatment on the Secondary Outcome Variables: CAVI, HRV, PhA, and Fat Mass

Table S5: Effects of Treatment on the Secondary Outcome Variables: BEPSI-K, BFI, FSS, and SF-36

Table S1: Baseline Characteristics of the Study Participants

Characteristic		ERW (n=29)	MW (n=24)	*P* value
**Demographic**	years			
Age		39.3±8.9	41.0±9.3	0.523*
Sex	no. (%)			1.000†
Female		25 (86.2%)	21(87.5%)	
Male		4 (13.8%)	3 (12.5%)	
Marital status	no. (%)			0.378‡
Married		18 (62.1%)	12 (50.0%)	
Single		11 (37.9%)	12 (50.0%)	
Educational level	no. (%)			0.701§
High school		3 (10.3%)	2 (8.3%)	
College/University		21 (72.4%)	17 (70.8%)	
Postgraduate		5 (17.2%)	5 (20.8%)	
Occupation	no. (%)			0.886§
Professionals		6 (20.7%)	5 (20.8%)	
White collar		20 (69.0%)	17 (70.8%)	
Blue collar		3 (10.3%)	2 (8.3%)	
**Clinical**				
d-ROMs	U.CARR	301.3 ± 68.6	347.3 ± 94.9	0.046*
BAP	μmol/L	2027.4 ± 226.9	2015.7 ± 274.8	0.734‖
TBARS (MDA)	μM	9.88 ± 4.67	8.90 ± 5.98	0.506*
8-OHdG	ng/mL	14.33 ± 6.88	10.90 ± 6.42	0.069*
oxLDL	U/L	52.43 ± 17.38	49.38 ± 13.61	0.520‖
GPx	nmol/min/mL	151.4 ± 31.1	140.1 ± 34.9	0.180‖
Body Mass Index	kg/m^2	22.2 ± 3.0	23.0 ± 3.1	0.357*
Blood pressure	mmHg			
Systolic		116.3 ± 10.6	116.3 ± 9.5	0.943†
Diastolic		72.0 ± 11.6	72.4 ± 9.5	0.971‖
Glucose	mg/dL	92.8 ± 7.5	92.3 ± 6.9	0.805*
AST	IU/L	20.0 ± 6.0	23.4 ± 17.4	0.872‖
ALT	IU/L	23.8 ± 46.3	22.1 ± 28.7	0.566‖
LDL-C	mg/dL	119.3 ± 30.9	111.8 ± 26.1	0.350*
TG	mg/dL	93.5 ± 59.4	104.5 ± 160.1	0.879‖

Note: Continuous variables are given as means ± standard deviations (SD) and compared by *T test or ‖Mann-Whitney U Test. Categorical variables are given as numbers (no) with percentages (%) and compared by ‡Pearson's Chi-Squared Test, †Fisher's Exact Test, or §Linear-by-Linear Association as appropriate. Abbreviations: ERW, Electrolyzed Reduced Water; MW, Mineral Water; d-ROMs, diacron-Reactive Oxygen Metabolites; BAP, Biological Antioxidant Potential; TBARS, Thiobarbituric Acid Reactive Substances; MDA, Malondialdehyde; 8-OHdG, 8-Hydroxy-2-Deoxyguanosine; oxLDL, Oxidized Low Density Lipoprotein; GPx, glutathione peroxidase; AST, Aspartate aminotransferase ; ALT, Alanine aminotransferase; LDL-C, Low-Density Lipoprotein Cholesterol; TG, Triglyceride.

Table S2: Effect of Treatment on the Primary Outcome Variables: Biomarkers of Oxidative Stress

	ERW (n=29)	MW (n=24)	Main Effects of Repeated Measures ANOVA				
Outcome Variables	Means±SD	Means±SD	Effect	F	df	*p*	pη2
d-ROMs	U.CARR						
Baseline	301.3±68.6	347.3±94.9	Time	6.61	1.68,85.65	0.004	0.115
4 wks	286.7±45.1	306.3±54.8	Group	8.04	1,51	0.007	0.136
8 wks	288.0±50.0	349.6±62.7	Time*Group	3.44	1.68,85.65	0.044	0.063
BAP	umol/L						
Baseline	2027.4±226.9	2015.7±274.8	Time	258.47	2,102	0.000	0.835
4 wks	2585.9±258.8	2504.8±187.8	Group	0.03	1,51	0.875	0.000
8 wks	2603.9±255.9	2670.4±180.2	Time*Group	3.20	2,102	0.045	0.059
TBARS (MDA)	μM						
Baseline	9.88±4.67	8.90±5.98	Time	6.23	2,102	0.003	0.109
4 wks	6.84±3.99	8.95±6.14	Group	0.06	1,51	0.809	0.001
8 wks	6.97±4.51	6.63±4.47	Time*Group	2.45	2,102	0.091	0.046
8-OHdG	ng/mL						
Baseline	14.33±6.88	10.90±6.42	Time	3.52	2,102	0.033	0.065
4 wks	13.87±8.31	15.63±11.18	Group	0.29	1,51	0.594	0.006
8 wks	11.87±7.90	10.87±7.00	Time*Group	2.02	2,102	0.138	0.038
oxLDL	U/L						
Baseline	52.43±17.38	49.38±13.61	Time	1.11	2,102	0.332	0.021
4 wks	53.60±15.32	52.00±12.94	Group	0.17	1,51	0.679	0.003
8 wks	52.24±15.97	52.1±12.99	Time*Group	0.63	2,102	0.534	0.012
GPx	nmol/min/mL						
Baseline	151.4±31.1	140.1±34.9	Time	27.07	2,102	0.000	0.347
4 wks	162.8±36.5	161.7±37.9	Group	0.22	1,51	0.639	0.004
8 wks	185.8±30.9	189.6±23.6	Time*Group	0.89	2,102	0.412	0.017

Note: *Interaction between Time and the Group. Abbreviations: ANOVA, Analysis of Variance; F, the F-value; df, degrees of freedom; p, statistical significance; pη2, partial eta squared; d-ROMs, diacron-Reactive Oxygen Metabolites; BAP, Biological Antioxidant Potential; TBARS, Thiobarbituric Acid Reactive Substances; MD A, Malondialdehyde; 8-OHdG, 8-Hydroxy-2-Deoxyguanosine; oxLDL, Oxidized Low-Density Lipoprote in; GPx, Glutathione Peroxidase.

Table S3: Effects of Treatment on the Secondary Outcome Variables: Biochemistry Parameters

	ERW (n=29)	MW (n=24)	Main Effects of Repeated Measures ANOVA				
Outcome Variables	Means ±SD	Means ±SD	Effect	F	df	*p*	pη2
NK Cell Activity	pg/mL						
Baseline	949.0±810.7	1263.4±905.2	Time	20.93	2,102	0.000	0.291
4wks	1943.5±1109.2	1829.0±1136.7	Group	0.40	1,51	0.528	0.008
8wks	1814.2±1091.8	2042.0±964.9	Time*Group	1.26	2,102	0.289	0.024
A.G.E.	AU						
Baseline	2.05±0.26	2.04±0.27	Time	20.94	2,102	0.000	0.291
4wks	1.83±0.32	1.82±0.23	Group	0.09	1,51	0.767	0.002
8wks	1.82±0.34	1.91±0.33	Time*Group	1.42	2,102	0.246	0.027
Glucose	mg/dL						
Baseline	92.8±7.5	92.3±6.9	Time	0.02	2,102	0.978	0.000
4wks	92.8±8.2	92.1±6.0	Group	0.22	1,51	0.640	0.004
8wks	93.0±5.9	91.8±6.2	Time*Group	0.09	2,102	0.916	0.002
HbA1c	mg/dL						
Baseline	5.23±0.29	5.32±0.30	Time	15.47	1.47,74.85	0.000	0.233
4wks	5.33±0.29	5.43±0.24	Group	1.49	1,51	0.228	0.028
8wks	5.27±0.27	5.36±0.25	Time*Group	0.01	1.47,74.85	0.971	0.000
Insulin	uU/mL						
Baseline	8.65±3.78	8.21±10.31	Time	2.57	1.71,87.32	0.091	0.048
4wks	6.66±3.19	7.18±4.76	Group	0.12	1,51	0.729	0.002
8wks	7.15± 3.19	8.50±6.78	Time*Group	0.89	1.71,87.32	0.401	0.017
HOMA-IR							
Baseline	2.21±0.93	1.95±2.74	Time	2.35	1.61,81.94	0.112	0.044
4wks	1.55± 0.80	1.65±1.15	Group	0.11	1,51	0.739	0.002
8wks	1.68± 0.80	1.98±1.61	Time*Group	0.49	1.61,81.94	0.575	0.009
Cortisol	ng/mL						
Baseline	80.81±35.53	81.78±25.30	Time	0.30	2,102	0.743	0.006
4wks	76.95±27.36	79.06±41.63	Group	0.60	1,51	0.443	0.012
8wks	72.63±27.47	85.70±26.27	Time*Group	1.19	2,102	0.308	0.023
TG	mg/dL						
Baseline	88.7±59.4	113.4±175.1	Time	0.72	1.28,65.05	0.432	0.014
4wks	88.5±64.8	94.5±66.1	Group	0.33	1,51	0.570	0.006
8wks	85.6± 61.6	90.3±57.8	Time*Group	0.49	1.28,65.05	0.530	0.010
LDL-C	mg/dL						
Baseline	119.3± 30.9	111.8±26.1	Time	2.71	2,102	0.071	0.051
4wks	120.4± 31.8	117.6±28.8	Group	0.24	1,51	0.628	0.005
8wks	114.6± 29.5	113.7±26.1	Time*Group	1.26	2,102	0.288	0.024

HDL -C	mg/dL						
Baseline	65.8±15.8	68.8±14.0	Time	0.16	2,102	0.855	0.003
4wks	66.1±15.8	69.6±16.0	Group	0.93	1,51	0.339	0.018
8wks	65.2±17.2	70.6±15.5	Time*Group	0.53	2,102	0.592	0.010
Lactic Acid	mmol/L						
Baseline	1.76± 0.86	1.52±0.55	Time	0.80	2,102	0.454	0.015
4wks	1.65± 0.65	1.58±0.61	Group	0.15	1,51	0.699	0.003
8wks	1.44± 0.45	1.61±0.47	Time*Group	2.43	2,102	0.093	0.045
Uric Acid	mg/dL						
Baseline	4.29±1.43	4.53±1.09	Time	2.63	2,102	0.077	0.049
4wks	4.26±1.33	4.37±1.20	Group	0.12	1,51	0.732	0.002
8wks	4.49±1.36	4.48±0.93	Time*Group	1.49	2,102	0.231	0.028
TB	mg/dL						
Baseline	0.72±0.38	0.77±0.26	Time	0.35	2,102	0.709	0.007
4wks	0.66±0.31	0.87±0.49	Group	1.47	1,51	0.231	0.028
8wks	0.70±0.36	0.76±0.36	Time*Group	2.01	2,102	0.140	0.038
GGT	IU/L						
Baseline	25.8±27.0	22.9±19.8	Time	0.82	1.36,69.49	0.403	0.016
4wks	24.0±20.4	31.3±44.9	Group	0.18	1,51	0.672	0.004
8wks	21.9±14.5	25.6±24.8	Time*Group	1.25	1.36,69.49	0.281	0.024
AST	IU/L						
Baseline	20.0±6.0	23.4±17.4	Time	1.75	1.69,86.07	0.180	0.033
4wks	19.1±6.1	25.3±23.9	Group	1.72	1,51	0.196	0.033
8wks	18.5±4.9	19.6±6.4	Time*Group	1.00	1.69,86.07	0.359	0.019
ALT	IU/L						
Baseline	23.8±46.3	22.1±28.7	Time	1.00	1.17,59.47	0.335	0.019
4wks	16.3±8.5	54.4±170.6	Group	0.31	1,51	0.020	1.041
8wks	16.3±7.8	20.2±26.1	Time*Group	1.48	1.17,59.47	0.232	0.028
ALP	IU/L						
Baseline	161.4±41.5	165.3±43.0	Time	2.76	2,102	0.068	0.051
4wks	159.9±43.5	170.3±56.4	Group	0.54	1,51	0.468	0.010
8wks	152.2±38.3	163.7±44.0	Time*Group	0.85	2,102	0.432	0.016
Serum Ca	mg/dL						
Baseline	9.00±0.28	9.05±0.27	Time	2.87	2,102	0.062	0.053
4wks	9.08±0.29	9.19±0.31	Group	2.04	1,51	0.159	0.039
8wks	9.05±0.34	9.19±0.45	Time*Group	0.35	2,102	0.707	0.007
Urine Ca	mg/dL						
Baseline	13.08±5.73	11.07±7.72	Time	0.57	2,102	0.567	0.011
4wks	9.84±5.76	13.20±11.79	Group	0.04	1,51	0.848	0.001
8wks	10.94±8.13	10.69±10.84	Time*Group	2.69	2,102	0.073	0.050
NLR							
Baseline	1.90 ±0.81	1.95±0.56	Time	2.19	2,102	0.117	0.041

4wks	1.71±0.54	1.86±0.47	Group	0.34	1,51	0.561	0.007
8wks	1.92±0.84	1.98±0.51	Time*Group	0.23	2,102	0.799	0.004
CRP	mg/dL						
Baseline	0.07±0.13	0.06±0.06	Time	0.02	1.51,77.01	0.952	0.000
4wks	0.08±0.20	0.05±0.06	Group	0.24	1,51	0.628	0.005
8wks	0.05±0.04	0.07±0.06	Time*Group	1.15	1.51,77.01	0.309	0.022

Note: *Interaction between Time and the Group. Abbreviations: NK, Natural Killer; A.G.E., Advanced Glycation End Products; HbA1c, Glycosylated Hemoglobin; HOMA-IR, Homeostatic Model Assessment for Insulin Resistance; AU, Arbitrary Units; TG, Triglyceride; LDL-C, Low-Density Lipoprotein Cholesterol; HDL-C, High-Density Lipoprotein Cholesterol; TB, Total Bilirubin; IU, International Unit; GGT, Gamma-Glutamyltransferase; AST, Aspartate Aminotransferase ; ALT, Alanine Aminotransferase; ALP, Alkaline Phosphatase; NLR, Neutrophil-Lymphocyte Ratio; CRP, C-Reactive Protein.

Table S4: Effects of Treatment on the Secondary Outcome Variables: CAVI, HRV, PhA, and Fat Mass

	ERW Group (n=29)	MW Group (n=24)		Main Effects of Repeated Measures ANOVA			
Outcome Variables	Means±SD	Means±SD	Effect	F	df	*p*	pη2
CAVI-Rt							
Baseline	6.44±0.80	6.36±1.06	Time	0.36	1.45, 73.80	0.627	0.007
4wks	6.32±0.80	6.36±0.73	Group	0.11	1,51	0.738	0.002
8wks	6.18±0.69	6.36±0.86	Time*Group	0.38	1.45, 73.80	0.615	0.007
CAVI-Lt							
Baseline	6.52±0.77	6.48±1.11	Time	0.73	1.44, 73.30	0.444	0.014
4wks	6.38±0.83	6.48±0.76	Group	0.32	1,51	0.577	0.006
8wks	6.23±0.69	6.42±0.81	Time*Group	0.29	1.44, 73.30	0.677	0.006
HRV-SDNN	ms						
Baseline	35.95±17.33	30.42±20.05	Time	0.93	2,102	0.399	0.018
4wks	34.05±15.26	35.33±13.47	Group	0.29	1,51	0.590	0.006
8wks	36.99±17.06	37.82±11.28	Time*Group	0.17	2,102	0.845	0.003
HRV-RMSSD	ms						
Baseline	27.49±13.44	27.96±14.20	Time	0.06	2,102	0.939	0.001
4wks	26.74±14.40	29.65±18.59	Group	0.49	1,51	0.487	0.010
8wks	26.03±12.03	28.72±13.04	Time*Group	0.17	2,102	0.845	0.003
HRV-PSI							
Baseline	71.92±81.46	52.85±31.23	Time	0.16	1.77, 90.31	0.825	0.003
4wks	71.54±59.39	54.99±33.62	Group	1.83	1,51	0.182	0.035
8wks	63.09±41.19	54.68±38.06	Time*Group	0.23	1.77, 90.31	0.765	0.005
HRV-TP	ms^2						
Baseline	913.43±613.60	1071.63±876.97	Time	0.69	2,102	0.502	0.013
4wks	985.86±1030.49	1041.21±1223.80	Group	0.07	1,51	0.795	0.001
8wks	1185.43± 1570.90	1115.73±973.82	Time*Group	0.16	2,102	0.851	0.003
HRV-LF/HF							
Baseline	1.52±1.30	2.01±2.14	Time	0.47	2,102	0.629	0.009
4wks	1.64±1.41	1.64±1.79	Group	0.00	1,51	0.958	0.000
8wks	2.19±2.43	1.64±1.34	Time*Group	1.65	2,102	0.197	0.031
Outcome	Means±SD	Means±SD	Effect	F	df	p	pη2

Variables							
Phase Angle	°						
Baseline	5.89±0.85	5.83±0.85	Time	15.74	1.75, 89.01	0.000	0.236
4wks	5.63±0.75	5.67±0.78	Group	0.00	1,51	0.952	0.000
8wks	5.62±0.67	5.60±0.84	Time*Group	0.53	1.75, 89.01	0.566	0.010
Reactance	Ω						
Baseline	58.20±6.77	57.34±8.65	Time	73.61	2,102	0.000	0.591
4wks	64.90±6.64	65.18±9.29	Group	0.26	1,51	0.610	0.005
8wks	65.83±6.82	63.40±7.79	Time*Group	2.05	2,102	0.135	0.039
Impedance	Ω						
Baseline	573.65±72.01	570.64±83.13	Time	364.15	2,102	0.000	0.877
4wks	669.27±85.20	666.11±97.96	Group	0.14	1,51	0.714	0.003
8wks	676.51±81.64	657.39±88.55	Time*Group	2.59	2,102	0.080	0.048
BMI	kg/m^2						
Baseline	22.23±3.11	23.03±3.12	Time	1.85	2,102	0.163	0.035
4wks	22.23±3.16	22.72±3.02	Group	0.60	1,51	0.443	0.012
8wks	22.20±3.12	22.88±2.90	Time*Group	1.70	2,102	0.189	0.032
Fat -Total	kg						
Baseline	16.21±4.88	17.30±5.03	Time	10.12	1.80, 91.60	0.000	0.166
4wks	16.18±5.00	16.55±4.90	Group	0.18	1,51	0.678	0.003
8wks	15.86±4.86	16.10±5.31	Time*Group	3.43	1.80, 91.60	0.041	0.063
Fat-Visceral	kg						
Baseline	1.86±0.96	2.00±0.95	Time	9.96	2,102	0.000	0.163
4wks	1.87±1.00	1.87±0.82	Group	0.04	1,51	0.846	0.001
8wks	1.79±0.94	1.80±0.93	Time*Group	3.76	2,102	0.027	0.069
Fat-Subcutaneous	kg						
Baseline	14.36±3.96	15.30±4.12	Time	9.81	1.78, 90.50	0.000	0.161
4wks	14.30±4.04	14.69±4.11	Group	0.22	1,51	0.644	0.004
8wks	14.07±3.96	14.30±4.41	Time*Group	3.29	1.78, 90.50	0.047	0.061

Note: *Interaction between Time and the Group. Abbreviations: CAVI, Cardio-Ankle Vascular Index; HRV, Heart Rate Variability; PSI, Physical Stress Index; SDNN, Standard Deviation of Normal to Normal Intervals; RMSSD, Root Mean Square of the Successive Differences; PSI, Physical Stress Index; TP, Total Power; LF, Low Frequency; HF, High Frequency; BMI, Body Mass Index.

Table S5: Effects of Treatment on the Secondary Outcome Variables: BEPSI-K, BFI, FSS, and SF-36

	ERW Group (n=29)	MW Group (n=24)	Main Effects of Repeated Measures ANOVA				
Outcome Variables	Mean±SD	Mean±SD	Effects	F	df	*p*	pη2
BEPSI-K							
Baseline	1.82± 0.65	1.73±0.51	Time	0.60	2,102	0.553	0.012
4wks	1.71± 0.72	1.73±0.51	Group	0.01	1,51	0.907	0.000
8wks	1.76± 0.73	1.78±0.56	Time*Group	0.68	2,102	0.511	0.013
BFI							
BFI Global							
Baseline	4.14±2.09	4.01±2.27	Time	4.28	2,102	0.016	0.077
4wks	3.60±1.98	3.56±2.09	Group	0.00	1,51	0.964	0.000
8wks	3.28±2.29	3.38±2.28	Time*Group	0.11	2,102	0.901	0.002
BFI Severity							
Baseline	5.69± 2.06	5.36±2.45	Time	3.11	2,102	0.049	0.057
4wks	5.49± 1.77	5.14±2.28	Group	0.33	1,51	0.566	0.007
8wks	4.90± 2.32	4.71±2.40	Time*Group	0.05	2,102	0.956	0.001
BFI Interference							
Baseline	3.36± 2.39	3.33±2.38	Time	3.72	2,102	0.028	0.068
4wks	2.66± 2.37	2.76±2.30	Group	0.04	1,51	0.845	0.001
8wks	2.47± 2.46	2.72±2.41	Time*Group	0.11	2,102	0.895	0.002
FSS							
Baseline	3.58± 1.58	3.65±1.68	Time	1.83	1.74, 88.48	0.172	0.035
4wks	3.15± 1.53	3.40±1.67	Group	0.01	1,51	0.928	0.000
8wks	3.39± 1.81	3.17±1.65	Time*Group	0.68	1.74, 88.48	0.489	0.013
Outcome Variables	Mean±SD	Mean±SD		F	df	*p*	pη2
SF-36 Health Survey							
Physical function (PF)							
Baseline	84.14±21.34	84.79±16.12	Time	0.24	2,102	0.785	0.005
4wks	84.83±14.42	80.83±21.30	Group	0.02	1,51	0.884	0.000
8wks	83.79±20.07	85.42±16.08	Time*Group	0.56	2,102	0.572	0.011
Role-Physical (RP)							
Baseline	70.69±35.40	71.88±28.85	Time	0.58	2,102	0.562	0.011
4wks	67.24±36.66	79.17±25.18	Group	0.75	1,51	0.389	0.015
8wks	71.55±35.81	79.17±31.85	Time*Group	1.02	2,102	0.365	0.020
Bodily Pain (BP)							
Baseline	70.78±23.49	76.88±16.83	Time	0.66	2,102	0.518	0.013
4wks	75.60±24.88	77.60±22.49	Group	0.11	1,51	0.747	0.002

8wks	78.71±22.50	75.42±19.46	Time*Group	1.20	2,102	0.307	0.023
General Health (GH)							
Baseline	53.97±18.00	58.75±19.69	Time	0.04	2,102	0.963	0.001
4wks	57.24±15.96	55.63±22.52	Group	0.08	1,51	0.780	0.002
8wks	56.35±18.02	57.29±21.47	Time*Group	1.62	2,102	0.203	0.031
Vitality (VT)							
Baseline	50.17±14.67	56.25±15.90	Time	1.00	2,102	0.371	0.019
4wks	51.21±15.85	53.13±16.14	Group	1.17	1,51	0.285	0.022
8wks	52.76±18.20	57.50±19.73	Time*Group	0.50	2,102	0.608	0.010
Social Functioning (SF)							
Baseline	74.57±20.45	82.81±16.41	Time	0.92	2,102	0.401	0.018
4wks	76.30±19.29	79.69±20.46	Group	1.18	1,51	0.283	0.023
8wks	79.97±22.61	82.71±14.82	Time*Group	0.67	2,102	0.514	0.013
Role-Emotional (RE)							
Baseline	65.52±44.08	79.17±33.79	Time	0.25	2,102	0.778	0.005
4wks	66.67±39.85	80.56±35.33	Group	1.37	1,51	0.248	0.026
8wks	72.41±36.81	77.79±33.57	Time*Group	0.78	2,102	0.463	0.015
Mental Health (MH)							
Baseline	63.72±16.07	67.00±13.36	Time	0.94	2,102	0.395	0.018
4wks	64.41±16.01	62.50±16.38	Group	0.24	1,51	0.628	0.005
8wks	63.90± 19.95	68.33± 16.72	Time*Group	1.43	2,102	0.245	0.027

Note: *Interaction between Time and the Group. Abbreviations: BEPSI-K, Brief Encounter Psychosocial Instrument - Korean version; BFI, Brief Fatigue Inventory; FSS, Fatigue Severity Scale; SF-36, 36-Item Short Form Survey.
